# Alternative Splicing Dynamics During the Lifecycle of *Salvia miltiorrhiza* Root Revealed the Fine Tuning in Root Development and Ingredients Biosynthesis

**DOI:** 10.3389/fpls.2021.797697

**Published:** 2022-01-21

**Authors:** Yajing Li, Peng Di, Jingfu Tan, Weixu Chen, Junfeng Chen, Wansheng Chen

**Affiliations:** ^1^Department of Pharmacy, Changzheng Hospital, Second Military Medical University, Shanghai, China; ^2^Center of Chinese Traditional Medicine Resources and Biotechnology, Institute of Chinese Materia Medica, Shanghai University of Traditional Chinese Medicine, Shanghai, China; ^3^State Local Joint Engineering Research Center of Ginseng Breeding and Application, College of Chinese Medicinal Materials, Jilin Agricultural University, Changchun, China; ^4^Shangyao Huayu (Linyi) Traditional Chinese Resources Co. Ltd., Linyi, China

**Keywords:** alternative splicing, *Salvia miltiorrhiza*, root development, life cycle, ingredients biosynthesis

## Abstract

Alternative splicing (AS) is an essential post-transcriptional process that enhances the coding and regulatory potential of the genome, thereby strongly influencing multiple plant physiology processes, such as metabolic biosynthesis. To explore how AS affects the root development and synthesis of tanshinones and phenolic acid pathways in *Salvia miltiorrhiza* roots, we investigated the dynamic landscape of AS events in *S. miltiorrhiza* roots during an annual life history. Temporal profiling represented a distinct temporal variation of AS during the entire development stages, showing the most abundant AS events at the early seedling stage (ES stage) and troughs in 45 days after germination (DAG) and 120 DAG. Gene ontology (GO) analysis indicated that physiological and molecular events, such as lateral root formation, gravity response, RNA splicing regulation, and mitogen-activated protein kinase (MAPK) cascade, were greatly affected by AS at the ES stage. AS events were identified in the tanshinones and phenolic acids pathways as well, especially for the genes for the branch points of the pathways as SmRAS and SmKSL1. Fifteen Ser/Arg-rich (SR) proteins and eight phosphokinases (PKs) were identified with high transcription levels at the ES stage, showing their regulatory roles for the high frequency of AS in this stage. Simultaneously, a co-expression network that includes 521 highly expressed AS genes, SRs, and PKs, provides deeper insight into the mechanism for the variable programming of AS.

## Introduction

Alternative splicing (AS) is a pivotal precursor mRNA (pre-mRNA) regulatory process, by which the two or more different mRNAs are produced from a single gene locus, which dramatically improves the complexity and flexibility of the transcriptome and proteome (Reddy, 2004; Reddy et al., 2012; Li et al., 2017; Laloum et al., 2018). In AS process, spliceosomes are usually recruited by splicing factors that combine with the 3′ and 5′ splice sites, then completing splice by two successive transesterification reactions (Zhang et al., 2010; Shang et al., 2017). It typically leads to four types of AS events: alternative 3′splice site choice (Alt.3′), alternative 5′ splice site choice (Alt.5′), exon skipping (ES), and intron retention (IR). In contrast to events in animals with ES as the most prevalent AS type, IR is the most common type in plants (Simpson et al., 2008; Shen et al., 2014). The first confirmation for the markedness of AS in plant development came from differential expression of Ser/Arg-rich (SR) protein splicing factors in different organs and development stages (Reddy et al., 2013). As molecular adaptors, SR proteins modulate the constitutive and AS of pre-mRNAs, which play critical roles in the RNA metabolism of higher eukaryotes (Palusa and Reddy, 2010). They are characterized by the presence of one or two terminal RNA recognition motif (RRM) and a *C*-terminal domain rich in arginine-serine dipeptides (RS domain) (Rosenkranz et al., 2021). The RRM motif could recognize and bind with the target sequence, the RS domain is involved in the interaction between proteins, sub-nuclear location, and regulation of RNA binding, modulated by the phosphorylation status (Ngo et al., 2005; Palusa et al., 2007; Sahebi et al., 2016).

Alternative splicing has been shown to influence plant growth, development, signal transduction, and the response to various situations (Simpson et al., 2008; Reddy et al., 2013). Due to rapidly occurring AS in response to cold, hundreds of genes showed changes in expression, such as numerous novel cold-responsive transcription factors and splicing factors/RNA-binding proteins (Calixto et al., 2019). PtrWND1B, an No Apical Meristem/Arabidopsis Transcription Activation Factor/Cup-shaped Cotyledon (NAC) domain transcription factor associated with black cottonwood (*Populus trichocarpa*) wood, produced two isoforms by AS, which played antagonistic roles in regulating cell wall thickening during fiber cell differentiation in Populus spp. It indicated that AS may enable more specific regulation of processes, such as fiber cell wall thickening during wood formation (Zhao et al., 2014). Besides, AS is also involved in the synthesis and regulation of plant secondary metabolites. Significant differences emerged for SlAN2like, an R2R3 MYB transcription factor-encoding gene has ability to accumulate anthocyanins in fruit peel, with splicing mutations determining a complete loss of function of the wild-type protein (Colanero et al., 2020). AS of EsMYBA1 resulted in three transcripts, two of them encode a MYB-related protein, which could interact with several bHLH regulators to activate the promoters of dihydroflavonol 4-reductase and anthocyanidin synthase (Huang et al., 2013).

*Salvia miltiorrhiza* (*S. miltiorrhiza;* Danshen in Chinese), a well-known member of the Labiatae family, has significant medicinal values, published genome data, massive RNA-seq, and metabolism libraries and is emerging as a model plant system for studying the regulation of secondary metabolites (Xu et al., 2016; Zhou et al., 2016; Du et al., 2018; Wu et al., 2018; Ma et al., 2021). The dried roots and rhizomes are medicinal parts harvested in spring or autumn. The main active components hydrophilic salvianolic acids (SAs) and components accumulate during the whole development process, especially in the later developmental stages. Current research studies that involve *S. miltiorrhiza* mainly focused on the discovery of new functional enzymes and transcription factors. However, related pre-mRNA regulation studies are lacking systematic characterization and lagging behind compared with model plants. There is only one report related to AS analysis in *S. miltiorrhiza*. Based on the next-generation and single molecular real-time sequencing of *S. miltiorrhiza* root transcriptome at maturity, four enzyme-coding genes involved in the biosynthesis of rosmarinic acid were found to undergo AS process (Xu et al., 2016). However, the AS landscape of *S. miltiorrhiza* during its entire developmental stages remains unexplored.

To investigate genome-wide AS events during its annual lifecycle of *S. miltiorrhiza*, transcriptomes of 36 samples from twelve developmental stages were examined by Illumina-based RNA-seq. AS landscape in the lifecycle of *S. miltiorrhiza* showed variability and flexibility accompanied by different developmental states and 521 active AS genes were selected. Then, thirteen genes related to the two-class active substances biosynthesis were predicted experience of AS. In particular, a potential regulatory network was constructed to characterize the associations among splicing factors, PKs, and the active AS genes. In conclusion, these results highlight the importance of AS during the life-cycle of *S. miltiorrhiza* and provide a new perspective and value database for plant growth regulation.

## Results

### The Alternative Splicing Landscape in *Salvia miltiorrhiza* Roots During Its Annual Lifecycle

**FIGURE 1:**
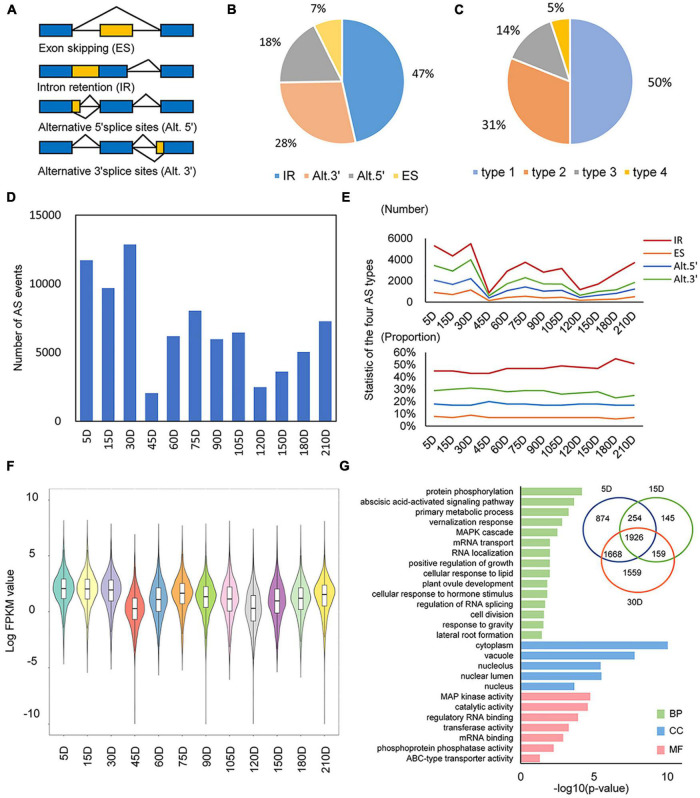
AS dynamics during the life-cycle of *Salvia miltiorrhiza*. **(A)** The representative forms of alternative splicing (AS), namely, exon skipping (ES), intron retention (IR), alternative 5′ splice sites (Alt.5′), and alternative 3′ splice sites (Alt.3′). **(B)** The proportion of each type of alternative splicing event. **(C)** The proportion of genes with multiple alternative splicing types. **(D)** Numbers of AS events in temporal dynamics. **(E)** Number and proportion of four types of AS events in temporal dynamics. **(F)** Violin plot of all expressed AS genes in the whole developmental stages. **(G)** Most significantly enriched GO terms for the first three period genes. Bar plots of –log_10_ transformed *p* are shown. BP, biological process; CC, cellular component; MF, molecular function.

We studied the AS dynamic of *S. miltiorrhiza* during an annual lifecycle [from 5 days after germination (DAG) to fruit stage] according to a temporal transcriptional profiling of *S. miltiorrhiza* roots. All samples were classified into four successive developmental stages as a seedling (5–30 DAG), elongation (45–75 DAG), swelling (90–120 DAG), and wilting (150–210 DAG), respectively. SpliceGrapher was performed to detect AS (Rogers et al., 2012). In total, 81,318 AS events were identified to distribute among 14,885 loci, which accounted for 56% of all loci in the *S. miltiorrhiza* genome. There were four major AS types identified (Figure 1A), with IR representing the most prevalent (51%), followed by Alt.3′ (28%), Alt.5′ (18%), and ES (3%) (Figure 1B). This result is consistent with findings in other plant species, such as *Arabidopsis thaliana* (Sugliani et al., 2010), soybean (Shen et al., 2014), and maize (Thatcher et al., 2016). In addition, half of the loci showed to undergo multiple AS types, showing that 5% of the genes went through four types of AS, 14% underwent three types, 31% experienced two types, and the rest (50%) showed just one type (Figure 1C).

We further analyzed the dynamic change of AS, showing a significant verified accumulation of AS events in different developmental stages of *S. miltiorrhiza* roots. The frequency of AS was first conducted in each sample (Figures 1D,E). As a result, ASs were abundant in the early seeding (ES) stage (5–30 DAG). Notably, AS counts showed a dramatic decrease in samples of 45 and 120 DAG, respectively. To analyze all four types of AS separately, they all showed similar enrichment trends and represented a consistent proportion (Figure 1E). To explore the origin of AS variation, the general transcription level and the number of transcripts were analyzed. As expected, the fewer transcripts were detected in samples of 45, 120, and 150 DAG (Supplementary Table 1) with the lower overall transcription level (Figure 1F). Besides, the variation trends of AS number were consistent with that of transcripts during the whole developmental stages of *S. miltiorrhiza* roots, indicating the positive correlation between AS and the transcription landscape.

The statistical results demonstrated that AS was abundant in the ES processes, indicating its essential regulation role in these developmental stages. GO enrichment was performed to reveal the major functions that AS contributes (Figure 1G). A total of 6,585 AS genes were identified at the ES stage. Their GO terms were mainly related to early root development and splicing mechanisms, such as lateral root formation (GO:0010311), response to gravity (GO:0009629), regulation of RNA splicing (GO:0043484), and MAPK cascade (GO:0000165) (Figure 1G). Among those AS genes, some have homologies that execute their regulating functions *via* AS forms. For example, AUXIN RESPONSE FACTOR7 (ARF7) from *A. thaliana* is spliced into three splicing variants to control lateral root formation (Zhang F. et al., 2020). The AS events of its homology in *S. miltiorrhiza* were identified, indicating its similar functional role in lateral root development.

### Genes With Alternative Splicing Switching at Specific Developmental Stages

**FIGURE 2:**
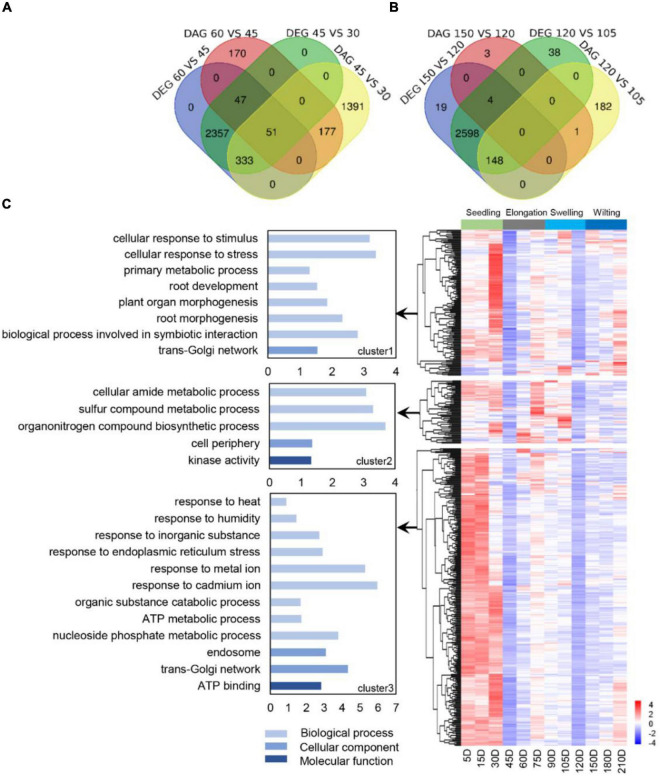
Selection and annotation of active AS genes relevant to splicing switch. **(A)** The Venn diagram of differentially expressed genes (DEG) and differentially spliced genes (DAS) genes at developmental nodes before and after 45 DAG and 120 DAG **(B)**. **(C)** Hierarchical clustering and GO enrichment of 521 active AS. The active genes showed three co-expressed modules. The z-score scale represents the mean-subtracted regularized log-transformed FPKM. AS, alternative splicing; GO, gene oncology.

The AS profiling presented two stages with a sharp decrease of AS at 45 and 120 DAG, respectively, suggesting two distinct stages for exploring the major physiological functions that AS involved. We compared the differential genes with AS and their GO terms between 45 DAG, 120 DAG, and their immediate stages (Figure 2). In total, 521 genes with AS and differently expressed were identified in both two stages (Figures 2A,B), which were grouped into three clusters according to their transcription profiling (Figure 2C). GO enrichment was further employed to investigate the function categories of each cluster, showing that cluster1 was mainly consisted of genes that involved in the biological processes responses to stress and root development, as a cellular response to stress (GO:0033554), the biological process involved in symbiotic interaction (GO:0044403), root morphogenesis (GO:0010015), and plant organ morphogenesis (GO:1905392). In cluster2, GO terms involved in the organonitrogen compound biosynthetic process (GO:1901566), sulfur compound metabolic process (GO:0006790), and cellular amide metabolic process (GO:0043603) had the most significant enrichment. Genes in cluster3 fell into the terms response to endoplasmic reticulum stress, inorganic substance, humidity, and heat (GO:0034976, GO:0071241, GO:0009270 and GO:0009408). Taken together, these selected active AS genes were probably to regulate the root development and coordinate the external clues through AS switching, which will provide potential targets for the development regulation of *S. miltiorrhiza*.

### Alternative Splicing Involved in the Biosynthesis Pathway of Active Ingredients of *Salvia miltiorrhiza*

**FIGURE 3:**
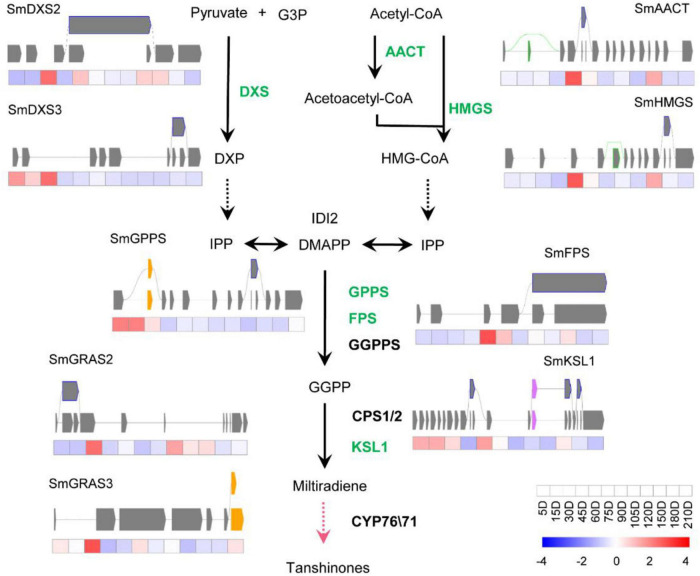
Transcript levels and alternative splicing isoforms of tanshinones biosynthetic genes and TFs. The green font means the gene has undergone AS process. The yellow boxes, purple boxes, blue frames, and green frames indicate Alt.3′, Alt.5′, IR, and ES splicing patterns, respectively. The black lines indicate the known enzymes, the pink line indicates the unknown enzymes. The gay lines indicate introns, gray squares represent exons. Alternative splicing.

**FIGURE 4:**
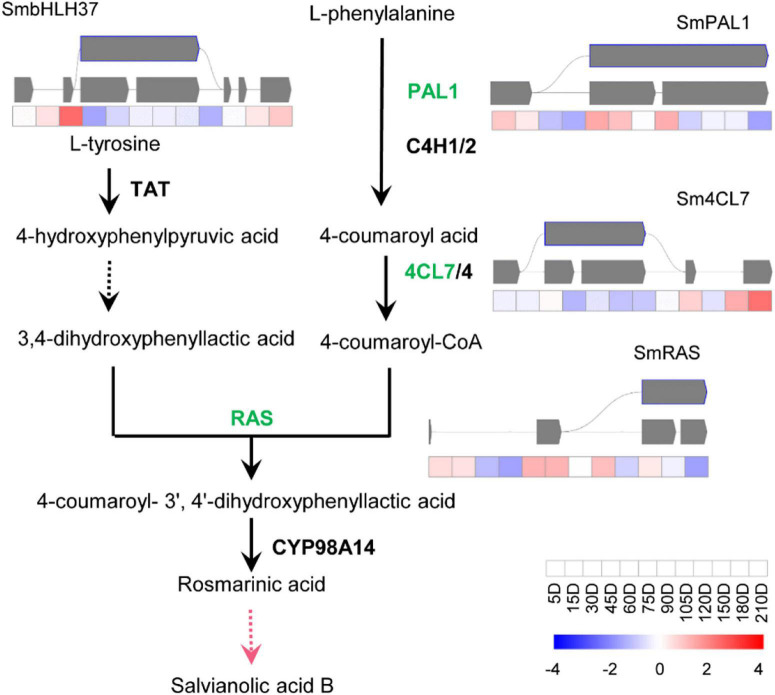
Transcript levels and alternative splicing isoforms of RA biosynthetic genes and TF. The yellow boxes, purple boxes, blue frames, and green frames indicate Alt.3′, Alt.5′, IR, and ES splicing patterns, respectively. The black lines indicate the known enzymes, the pink line indicates the unknown enzyme. The gay lines indicate introns, gray squares represent exons.

To study how AS influences the biosynthesis of effective metabolites of *S. miltiorrhiza*, the AS of genes involved in the biosynthetic pathways of tanshinones and SAs were analyzed, i.e., the genes coding catalytic enzymes and known transcriptional factors (TFs). Totally, thirteen genes were shown to undergo the AS process, i.e., ten catalytic genes and three TFs. There were seven catalytic genes and two TFs of the tanshinone pathway (Figure 3), and three pathway genes, and one TF in the SA pathway (Figure 4), most of which were in IR splicing pattern. To verify the AS we identified, they were compared to the AS that was previously identified according to a full-length transcriptome of *S. miltiorrhiza* (Xu et al., 2016). 4CL7 was spliced through IR, which is consistent with the previous finding. Whereas, the other AS events represented in this study were not identified. The differences might be due to the different developmental stages of roots or the genetic variation of *S. miltiorrhiza* accessions.

### Identification and Phylogenetic Analysis of Ser/Arg-Rich Genes in *Salvia miltiorrhiza*

**FIGURE 5:**
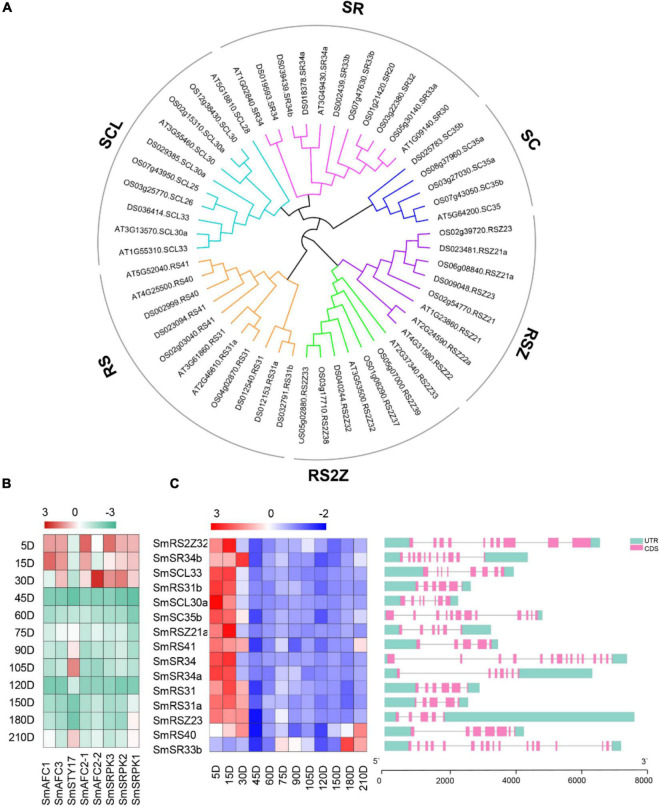
Identification of SR proteins and phosphokinases in *S. miltiorrhiza* genome. **(A)** Phylogenetic analysis of SR proteins from *Arabidopsis*, rice, and *S. miltiorrhiza* using the complete protein sequences. Alignments of 52 SR proteins from *A. thaliana*, rice, and *S. miltiorrhiza* were performed and the Neighbor-joining tree was constructed using MEGA 7.0 software. **(B)** The transcript levels of eight PKs. **(C)** The transcript levels of SR proteins coding genes and gene structure analyses were performed by TB tools software. The green boxes, pink boxes, and black lines indicate upstream/downstream UTR, exons, and introns, respectively.

**FIGURE 6:**
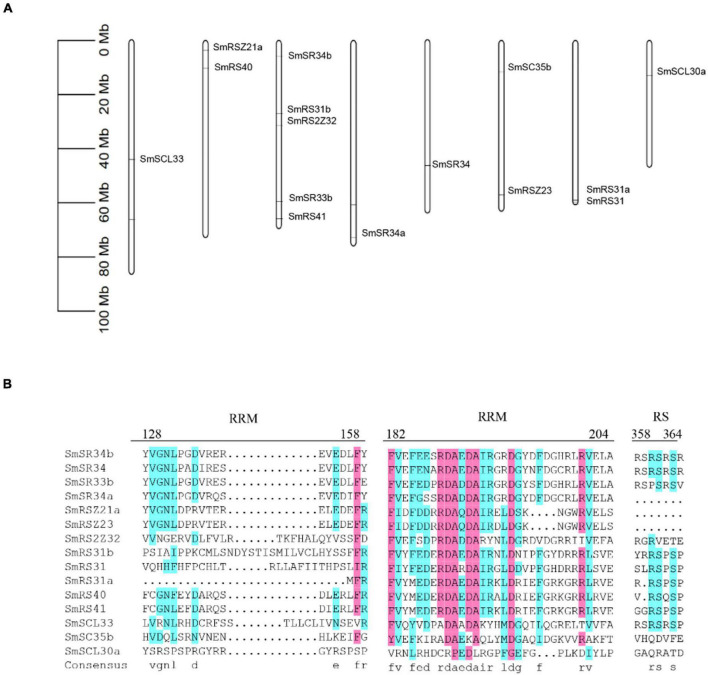
Chromosomal distribution and multiple sequence alignment of SR proteins. **(A)** Chromosomal distribution of SR proteins coding genes. **(B)** Multiple sequence alignment was obtained by the DNAMAN software. The family-specific conserved motifs were indicated.

Ser/Arg-rich is one of the determinants of splicing patterns (Kalyna et al., 2006; Zhou and Fu, 2013; Sahebi et al., 2016). We identified SR proteins from the *S. miltiorrhiza* genome. Totally, fifteen SR coding genes were identified with predicted peptides length ranging from 156 to 630 amino acids (Supplementary Table 2). The chromosomal distribution map showed that the fifteen SR proteins were successfully mapped to 8 chromosomes (Figure 6A). Chr3 contained five SR proteins, chr2, chr6, and chr7 contained two SR proteins, and the other four chromosomes contained one SR protein. They were clustered into six sub-groups according to a phylogenetic tree that includes 52 SR proteins (18 in *A. thaliana*, and 19 in *Oryza sativa*) (Figure 5A), which was consistent with the known sub-families in plants (SCL, SR, SC, RSZ, RS2Z, and RS) (Rosenkranz et al., 2021). All SR genes showed variant gene structures with different introns numbers from 4 to 14. Multiple sequence alignment of these SR proteins showed that all of them contained at least one RRM, for example, the conserved ribonucleoprotein-type (RNP-type) RNA-binding domains “GFAFVEFEDPRDAEDA,” “SWQDLKD,” and “RGG.” Overall, their expressions in the ES stage were higher compared with other periods, which was consistent with the highest abundance of AS events in these periods (Figure 5C).

The phosphorylation/dephosphorylation cycle of SR proteins is linked to all of their activities in the cell, such as spliceosome assembly, splice site selection, protein-interacting, and RNA-binding properties (Zhou and Fu, 2013). The Clk/Sty family members were phosphorylated and interacted with SR proteins and other splicing factors to participate in the modulation of pre-mRNA splicing (Reddy, 2007). The SR protein kinases (SRPKs) could phosphorylate SR proteins with outstanding efficiency and specificity (Ngo et al., 2005). Herein, three SRPKs and five Clk/Stys were selected according to genome annotation. The trend of their transcript levels was consistent with that of SR proteins, which was higher in the ES stages, and lower in 45 and 120 DAG (Figure 5B).

### Network of Ser/Arg-Rich Splicing Factors, Phosphokinases, and the Active Alternative Splicing Genes

**FIGURE 7:**
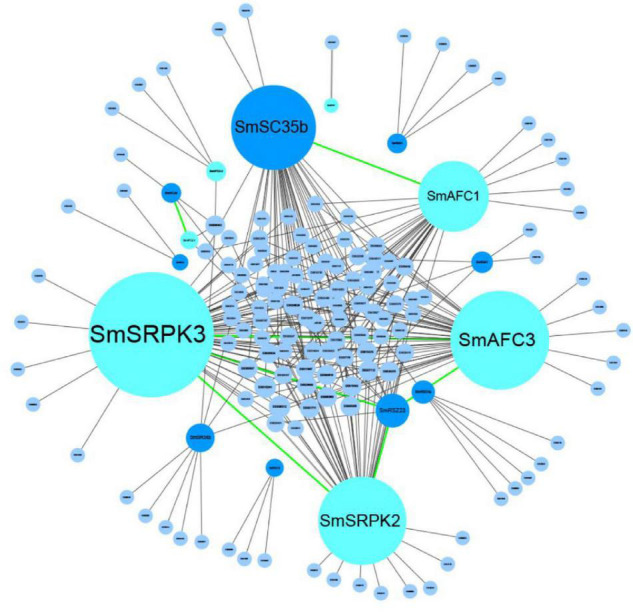
The potential regulatory network of SR splicing factors, phosphokinase, and AS genes. The gray lines represent the correlation between SR proteins or PKs and the active genes. The green lines represent the correlation between PKs and SR proteins or PKs. AS, alternative splicing.

To determine potential associations between the 521 active AS genes, SR splicing factors, and PKs, we constructed a network based on the Spearman correlation of their transcriptional levels. Significant correlations (correlations > 0.95, *p* < 0.05) were summarized in the network (Figure 7 and Supplementary Table 3). The results showed that nine SR proteins and seven PKs were significantly associated with 140 active AS genes, and all represented a positive correlation. Splicing factors SmSC35b had the most significant correlation with 39 AS genes. The SmSRPK3, SmAFC3, SmSRPK2, and SmAFC1 were the PKs most frequently associated with the active AS genes, accounting for 61, 47, 41, and 32 AS genes, respectively. Besides, ASs of SmSRPK2 and SmSRPK1 were identified as well, showing the interaction between AS processes and PKs.

Of note, SmSC35b was associated with SmAFC1, and both of them are related to 22 active AS genes. It intimated that SmSC35b was probably phosphorylated by SmAFC1 and further regulated the AS processes of 22 target genes. The same situation occurred in SmSRPK3, SmAFC3, SmSRPK2, and SmRSZ23, implying SmRSZ23 was probably phosphorylated by SmSRPK3, SmAFC3, and SmSRPK2, and further regulated the AS processes of seven target genes. The above results provided targets for the further research of the mechanism of AS in *S. miltiorrhiza*.

## Discussion

Alternative splicing phenomenon is ubiquitous in eukaryote genomes, which offers the chance to generate novel protein variants from a single gene locus (Syed et al., 2012; Yang et al., 2014). Previous studies showed that AS played important roles in many biological processes, such as photosynthesis, defense responses, flowering timing, and stress responses of the model plant *A. thaliana* (Sugliani et al., 2010; Martin et al., 2021) and crops, such as soybean (Shen et al., 2014), cotton (Wang et al., 2018, 2019), and maize (Thatcher et al., 2016), but little reports on herbs. Here, we reported the AS dynamic landscape of *S. miltiorrhiza* root during the developmental process from seedling to maturation. Temporal transcriptome profiling revealed that AS displays malleability during its annual lifecycle. In agreement with results from large-scale studies in plants, IR was the most prominent among the four AS types (Gulledge et al., 2012; Syed et al., 2012; Eckardt, 2013; Zhou and Fu, 2013; Sahebi et al., 2016). Besides, there were more than half of *S. miltiorrhiza* AS genes experienced only a single type of AS event. This phenomenon was also reported in *Gossypium austral* (Feng et al., 2019), grapevine (Aleynova et al., 2020), and peanut (Ruan et al., 2018). It indicated that many AS genes prefer only one splicing type to regulate pre-mRNA expression patterns in plants. In particular, AS events occurred more in the early seedling stage and less in the late development stages, especially in 45 and 120 DAG. Although there were significant differences in the number of AS events, the ratios of the four AS types were stable overall. Splicing dynamics in the seed of *A. thaliana* during dehydration showed that gene expression level and AS had a slightly opposite trend. Overall transcription was reduced between 14 and 20 days after pollination, while AS was increased (Srinivasan et al., 2016). Here, we found the gene expression levels were consistent with the occurrence of AS events, which was high in the first three developmental stages and reached troughs at 45 DAG and 120 DAG. The dramatic decrease of AS and transcripts in these developmental stages might be correlated to the biomass allocation between above and below-ground organs of *S. miltiorrhiza* in certain developmental stages. For example, plants at 45 DAG in this study were in the initial stage of the reproductive growth study, indicating a biomass allocation shift toward the above part to form reproductive organs (Poorter et al., 2012; Mason et al., 2017). Relatively, a massive of biological processes in roots might be reduced, including the transcriptional aspect.

The early stages of seedling development are essential to execute the correct body plan and initiate a new reproductive cycle (Szakonyi and Duque, 2018). Plant physiological functions are activated in this process, for example, hypocotyls gradually elongate and lateral roots germinate. It is well known that hormones are important regulators of early plant development. Genes involved in auxin biosynthesis also have a role in root formation, from the initiation of a root meristem during embryogenesis to a functional root system with a primary root, secondary lateral root branches, and adventitious roots (Kriechbaumer et al., 2012; Olatunji et al., 2017). Low external abscisic acid (ABA) concentrations stimulated root growth of *A. thaliana* while high ABA concentrations inhibited it in drought stress (Lorkovic, 2009). There are complex cross-talking among different kinds of hormones, which regulate the development of plant roots precisely. Comparably, GO terms enrichment analysis revealed that functions of the AS genes in the ES stage of *S. miltiorrhiza* were mainly enriched in typical seedling physiological processes, such as cellular response to hormone stimulus (GO:0032870), response to gravity (GO:0009629), and lateral root formation (GO:0010311), indicating that AS is essential in the regulation of plant seedling development. Recently, a JA-inducible rice gene (OsPDR1) that encodes a member of the pleiotropic drug resistance (PDR) subfamily of ABC transporters was found to produce three splice isoforms. The three OsPDR1 transcripts were developmentally controlled and differentially regulated by jasmonates and pathogen infection. The OsPDR1.2- and OsPDR1.3-overexpressing plants exhibited higher JAs content and stronger growth inhibition and disease resistance than OsPDR1.1-overexpressing plants. These results indicated that AS affects the function of the OsPDR1 gene in the regulation of growth, development, and disease resistance (Zhang H. et al., 2020). In addition to the GO terms related to the AS mechanism, we also found that AS genes in the ES stage were significantly enriched in ABC-type transporter activity (GO:0140359). This indicated that the ABC family transporter proteins also participated in the growth regulation of the seedling stage undergone AS process in *S. miltiorrhiza*. Further study is needed to reveal the biological function of different isoforms.

The biosynthetic pathways of the two types of active ingredients can be divided into upstream pathways and downstream pathways. The upstream pathway forms a skeleton structure, and the downstream pathway is post-modified on the skeleton to form a cluster of abundant compound species. So far, the upstream pathways are basically clarified, but the gene functions of the downstream pathways are rarely reported. Studies mainly focused on the CYP76A and CYP71D subfamilies in the downstream pathway of tanshinones, and the CYP98A subfamily in phenolic acids. The content of the two types of compounds is lower in the seedling stage. With the growth and development of *S. miltiorrhiza*, the content gradually increases and accumulates. Here, ten catalytic genes and three TFs of *S. miltiorrhiza* were predicted to experience AS processes. All of them were upstream pathway genes, which participated in forming the framework of compounds, especially the SmKSL1 in the tanshinone pathway and SmRAS in the RA pathway. Except 4CL7, the other twelve genes were not identified in the known full-length transcriptome data of *S. miltiorrhiza* (Xu et al., 2016). They were very likely the new variations and mediated the two-class compounds biosynthesis pathway. Besides, they were also possibly caused by the short reads of next-generation sequencing and further identity was necessary.

Limited studies with plant SR proteins suggested pivotal roles in growth and development and plant responses to the environment. AtRSZ33 is expressed during embryogenesis and early stages of seedling formation, as well as in flower and root development. Ectopic expression of atRSZ33 caused pleiotropic changes in plant development, resulting in increased cell expansion, and polarization changes in cell elongation and division (Kalyna et al., 2003). The RS domain of *A. thaliana* splicing factor RRC1 is required for phytochrome B signal transduction (Shikata et al., 2012). Here, we identified fifteen SR proteins distributed in six sub-families from the genome of *S. miltiorrhiza*. Three SRPKs and five Clk/Stys were selected based on the genome annotation. Both of their expression tendencies were higher in the ES stage, which was consistent with the more AS events in this stage. Therefore, there was a tight regulatory network among AS events, SR proteins, and PKs.

The detection of prominent AS switches and development-specific splice events has endorsed an important regulatory function at the splicing level (Szakonyi and Duque, 2018; Li et al., 2020; Ganie and Reddy, 2021; Martin et al., 2021). Here, we screened 521 AS genes with significant splicing switches in the development process of *S. miltiorrhiza*. Their functions were mainly enriched in the biological processes related to plant development and responses to environmental clues, such as root morphogenesis and response to humidity. These genes were more active after the AS process to mediate the root growth and development. Further network analysis showed that there were 140 active AS genes significantly correlated to the expression of nine SR transcripts and seven PK transcripts. These target splicing factors and PK candidates will provide a basis for the research on the AS regulation mechanism of *S. miltiorrhiza.*

In summary, we characterized the AS dynamic landscape in an annual lifecycle of *S. miltiorrhiza*, selected 521 active AS genes, and predicted ten catalytic genes and three TFs undergo AS process. Then we identified the SR proteins genome-wide and constructed a network between the active AS genes, SR proteins, and PKs. It proposed a new perspective and reference data for the growth and development regulation of *S. miltiorrhiza*.

## Materials and Methods

### Plant Materials

*Salvia miltiorrhiza* plants were cultivated at the Zealquest Scientific Technology Co. Ltd. Roots were collected at 5, 15, 30, 45, 60, 75, 90, 105, 120, 150, 180, and 210 DAG, with three repeats for each developmental stage. The harvested samples were put into liquid nitrogen immediately and then stored in a refrigerator at −80°C for RNA-seq.

### mRNA-Seq and Alternatively Spliced Isoform Analysis

Paired-end libraries were sequenced by Illumina NovaSeq6000 sequencing (150 bp*2, Shanghai Biozeron Co., Ltd, Shanghai, China). The raw paired-end reads were trimmed and quality controlled by Trimmomatic with parameters. Then clean reads were separately aligned to reference genome with orientation mode using hisat2^1^ software with default parameters. The expression level for each gene was calculated using the fragments per kilobase of exon per million mapped reads (FRKM) method. R statistical package edgeR was used for differential expression analysis. Next, SpliceGrapher was used to detect AS events by taking gene models and RNA-seq alignments as input and converting detected splice isoforms into splice graphs (Rogers et al., 2012). Introns fully subsumed by an exon were labeled as intron retention. Overlapping exons that differed at their 5′ or 3′ splice junctions were considered as Alt.3′ or Alt.5′ splicing events, respectively. Finally, exons absent in other isoforms were considered as ES events.

### Ser/Arg-Rich Proteins Identification and Evolutionary Analyses

Protein sequences of AtSRs and OsSRs were downloaded from TAIR and JGI databases, respectively. Candidate SR genes were firstly acquired with The Basic Local Alignment Search Tool (BLAST) search from the Danshen genome database using *A. thaliana* and *Oryza sativa* SRs as queries. Subsequently, SR candidate genes were further checked with PFAM and CDD databases. All the protein sequences of three species were used to construct a neighbor-joining (NJ) phylogenetic tree using MEGA 7.0 software. ExPASy proteomics server was used to predict the molecular weight and isoelectric points (pI) of Danshen SR proteins^2^. The MEME program was used to identify the conserved proteins motifs^3^. Furthermore, all identified motifs were annotated according to InterProScan^4^, visualized genes structure with TBtools software (Chen et al., 2020).

### Screening of the Active Alternative Splicing Genes

Establish the parameters of each group according to whether the gene experienced AS and the number of AS events. If the gene did not occur AS at this period, the value was 0, and if two AS events occurred, it was 2. Then applied the rank-sum test with *p* < 0.05 as the standard to screen differential AS events. Simultaneously, the limma R package was used to screen differentially expressed genes with *p* < 0.05 and | logFC| > 1.5 as the screening criteria. Here, we defined the intersection of differentially expressed genes and differentially spliced genes as the important analysis targets.

### Construction of a Correlation Network

Spearman correlation tests were used to evaluate the relationships between the expression of splicing factor genes and PK and AS genes selected. A significant correlation was filtered with correlations > 0.95 and *p* < 0.05. Then, correlation plots were generated using Cytoscape (3.8.1).

## Data Availability Statement

The datasets presented in this study can be found in online repositories. The names of the repository/repositories and accession number(s) can be found below: http://bigd.big.ac.cn/, PRJCA006864.

## Author Contributions

WsC and JC were the leading investigators of this research program. JC designed the experiments. YL analyzed the data and wrote the manuscript. PD, WxC, and JT assisted in discussing the results. All authors contributed to the article and approved the submitted version.

## Conflict of Interest

JT and WxC were employed by the company Shangyao Huayu (Linyi) Traditional Chinese Resources Co. Ltd. The remaining authors declare that the research was conducted in the absence of any commercial or financial relationships that could be construed as a potential conflict of interest.

## Publisher’s Note

All claims expressed in this article are solely those of the authors and do not necessarily represent those of their affiliated organizations, or those of the publisher, the editors and the reviewers. Any product that may be evaluated in this article, or claim that may be made by its manufacturer, is not guaranteed or endorsed by the publisher.

## Footnotes

1
https://ccb.jhu.edu/software/hisat2/index.shtml

2
https://web.expasy.org/protparam/

3
http://meme-suite.org/tools/meme

4
http://www.ebi.ac.uk/interpro/
